# Roughness of (*ℤ*_+_, *ℤ*_−_)-Nonuniform Exponential Dichotomy for Difference Equations in Banach Spaces

**DOI:** 10.1155/2014/819046

**Published:** 2014-01-23

**Authors:** Nicolae Lupa

**Affiliations:** Department of Mathematics, “Politehnica” University of Timişoara, P-ţa Victoriei 2, 300006 Timişoara, Romania

## Abstract

In this paper we study the roughness of (*ℤ*_+_, *ℤ*_−_)-nonuniform exponential dichotomy for nonautonomous difference equations in the general context of infinite-dimensional spaces. An explicit form is given for each of the dichotomy constants of the perturbed equation in terms of the original ones. We emphasize that we do not assume any boundedness condition on the coefficients.

## 1. Introduction

The notion of uniform exponential dichotomy introduced by Perron in [1] for differential equations and by Li in [2] for difference equations plays an important role in the qualitative study of stable and unstable manifolds. In the nonautonomous setting, uniform exponential dichotomy is too restrictive and it is important to consider a more general behavior, for example, the nonuniform case, where a consistent contribution is due to Barreira and Valls [3, 4]. Their study is motivated by ergodic theory and nonuniform hyperbolic theory. We also refer to [5, 6].

One of the most important properties of exponential dichotomy (uniform or not) is its roughness (i.e., the manner in which exponential dichotomy varies under sufficiently small perturbations). The study of roughness of uniform exponential dichotomy has been intensively investigated (we mention in particular [7–9] and the references therein). For the roughness of nonuniform exponential dichotomy, we refer to [3, 10] for continuous time and to [4] for discrete time.

The aim of this paper is to extend a discrete version of a roughness result from [11] to infinite-dimensional spaces. Another difference here is that we consider the general case of nonuniform exponential dichotomies and we do not assume any boundedness condition on the coefficients.

## 2. Preliminary Definitions

As usual, *ℤ* denotes the ring of integers. Also, we denote by

(1) $$\begin{array}{c} {\mathbb{Z}}_{+}=\left\{m\in \mathbb{Z}:m\geq 0\right\},\;\;{\mathbb{Z}}_{-}=\left\{m\in \mathbb{Z}:m\leq 0\right\}. \end{array}$$

Let *X* be a real or complex Banach space and let *ℬ*(*X*) be the Banach algebra of all bounded linear operators on *X*. The norms on *X* and on *ℬ*(*X*) will be denoted by ||·||. For an arbitrary sequence (*a*_*m*_)_*m*∈*ℤ*_, in order to ease computation, we set by convention

(2) $$\begin{array}{c} \overset{m-1}{\underset{k=m}{\sum }}a_{k}=0,\;\;m\in \mathbb{Z}. \end{array}$$

In this paper, we consider the following nonautonomous linear difference equation:

(3) $$\begin{array}{c} x_{m+1}=A_{m}x_{m},\;m\in \mathbb{Z}, \end{array}$$

where (*A*_*m*_)_*m*∈*ℤ*_ ⊂ *ℬ*(*X*) is a sequence of invertible operators on the Banach space *X*. The corresponding (forward and backward) evolution operator of (3) is defined by

(4) $$\begin{array}{c} 𝒜\left(m,n\right)=\{\begin{array}{cc} A_{m-1}\cdots A_{n} & \text{for}\;m>n,\\ Id & \text{for}\;m=n,\\ A_{m}^{-1}\cdots A_{n-1}^{-1} & \text{for}\;m<n, \end{array} \end{array}$$

where *Id* is the identity operator on *X*. It is easy to see that

(5) $$\begin{array}{c} 𝒜\left(m,n\right)𝒜\left(n,p\right)=𝒜\left(m,p\right),\;\text{for}\;m,n,p\in \mathbb{Z}. \end{array}$$

Without the invertibility assumption on *A*_*m*_ ∈ *ℬ*(*X*), *m* ∈ *ℤ*, neither the backward solutions to (3), nor the evolution operator *𝒜*(*m*, *n*) exists for *m* < *n*.

**Definition 1**

We say that a sequence of projections *P*_*m*_ ∈ *ℬ*(*X*), *m* ∈ *ℤ* (i.e., *P*_*m*_^2^ = *P*_*m*_), is an *invariant projector* for (3) if

(6) $$\begin{array}{c} A_{m}P_{m}=P_{m+1}A_{m},\;\forall m\in \mathbb{Z}. \end{array}$$

If *P* = (*P*_*m*_)_*m*∈*ℤ*_ is a sequence of projections on *X*, then we denote by

(7) $$\begin{array}{c} Q_{m}=Id-P_{m} \end{array}$$

the complementary projection of *P*_*m*_, *m* ∈ *ℤ*. We notice that if *P* = (*P*_*m*_)_*m*∈*ℤ*_ is an invariant projector for (3), then *Q* = (*Q*_*m*_)_*m*∈*ℤ*_ is also an invariant projector for (3).

**Remark 2**

Relation (6) is equivalent to

(8) $$\begin{array}{c} 𝒜\left(m,n\right)P_{n}=P_{m}𝒜\left(m,n\right),\;\forall m,n\in \mathbb{Z}. \end{array}$$

**Definition 3 (see [4, pp. 3582])**

Equation (3) is said to have a *nonuniform exponential dichotomy* on *J*, where *J* is *ℤ*_+_, *ℤ*_−_ or *ℤ*, if there exist an invariant projector *P* = (*P*_*m*_)_*m*∈*ℤ*_ and constants *a*, *D* > 0 and *ɛ* ≥ 0 such that

 (*d*_1_) ||*𝒜*(*m*, *n*)*P*_*n*_|| ≤ *De* ^*ɛ*|*n*|^ *e* ^−*a*(*m*−*n*)^, for  *m* ≥ *n*  in  *J*;
 (*d*_2_) ||*𝒜*(*m*, *n*)*Q*_*n*_|| ≤ *De* ^*ɛ*|*n*|^ *e* ^−*a*(*n*−*m*)^, for  *m* ≤ *n*  in  *J*.

The constant *ɛ* measures the nonuniformity of the dichotomy. In particular, when *ɛ* = 0, (3) is said to have a uniform exponential dichotomy. The projector *P* = (*P*_*m*_)_*m*∈*ℤ*_ will be called the dichotomy projector.

**Definition 4**

We say that (3) has a (*ℤ*_+_, *ℤ*_−_)-*nonuniform exponential dichotomy* if there exist two invariant projectors *P*_+_ = (*P*_*m*_^+^)_*m*∈*ℤ*_ and *P*_−_ = (*P*_*m*_^−^)_*m*∈*ℤ*_ satisfying

(9) $$\begin{array}{c} P_{m}^{+}P_{m}^{-}=P_{m}^{-}P_{m}^{+}=P_{m}^{+},\;\forall m\in \mathbb{Z}, \end{array}$$

and there exist constants *a*, *D* > 0 and *ɛ* ≥ 0 such that the following estimates hold:

(*d*_1_^+^) ||*𝒜*(*m*, *n*)*P*_*n*_^+^|| ≤ *De* ^*ɛn*^ *e* ^−*a*(*m*−*n*)^, for  *m* ≥ *n* ≥ 0;
(*d*_2_^+^) ||*𝒜*(*m*, *n*)*Q*_*n*_^+^|| ≤ *De* ^*ɛn*^ *e* ^−*a*(*n*−*m*)^, for  *n* ≥ *m* ≥ 0;
(*d*_1_^−^) ||*𝒜*(*m*, *n*)*P*_*n*_^−^|| ≤ *De* ^*ɛ*|*n*|^ *e* ^−*a*(*m*−*n*)^, for  0 ≥ *m* ≥ *n*;
(*d*_2_^−^) ||*𝒜*(*m*, *n*)*Q*_*n*_^−^|| ≤ *De* ^*ɛ*|*n*|^ *e* ^−*a*(*n*−*m*)^, for  0 ≥ *n* ≥ *m*.

If *ɛ* = 0, then we say that (3) has a (*ℤ*_+_, *ℤ*_−_)-*uniform exponential dichotomy*. This concept of exponential dichotomy was studied in [11] for differential equations in finite-dimensional spaces and in [12] for reversible evolution families in Banach spaces.

Note that the existence of (*ℤ*_+_, *ℤ*_−_)-nonuniform exponential dichotomy is equivalent to the existence of nonuniform exponential dichotomy on both *ℤ*_+_ and *ℤ*_−_ with dichotomy projectors *P*_+_ and *P*_−_ satisfying relation (9). In the particular case when *P*_*m*_^+^ = *P*_*m*_^−^, for all *m* ∈ *ℤ*, we obtain the concept of nonuniform exponential dichotomy on *ℤ*. This means that if (3) has a nonuniform exponential dichotomy on *ℤ*, then it also has a (*ℤ*_+_, *ℤ*_−_)-nonuniform exponential dichotomy.

**Remark 5**

If (3) has a (*ℤ*_+_, *ℤ*_−_)-nonuniform exponential dichotomy, then we have that

(10) $$\begin{array}{c} ||P_{n}^{+}||\leq De^{ɛn},\;\text{for}\;n\geq 0,\\ ||P_{n}^{-}||\leq De^{ɛ|n|},\;\text{for}\;n\leq 0. \end{array}$$

Although relation (9) is replaced by

(11) $$\begin{array}{c} P_{m}^{+}P_{m}^{-}=P_{m}^{-}P_{m}^{+}=P_{m}^{-},\;\forall m\in \mathbb{Z}, \end{array}$$

the notion of (*ℤ*_+_, *ℤ*_−_)-nonuniform exponential dichotomy is different from the nonuniform version of exponential trichotomy defined in [13] for continuous time and in [14] for discrete time. This is due to the fact that no information is given regarding the boundedness of the projections *P*_*m*_^+^ for *m* ≤ 0 and *P*_*m*_^−^ for *m* ≥ 0 (for more details, we refer to [15]).

## 3. Roughness

In this section, we study the roughness of (*ℤ*_+_, *ℤ*_−_)-nonuniform exponential dichotomy for difference equations in Banach spaces. Thus, we investigate the existence of (*ℤ*_+_, *ℤ*_−_)-nonuniform exponential dichotomy for the perturbed linear difference equation

(12) $$\begin{array}{c} x_{m+1}=\left(A_{m}+B_{m}\right)x_{m},\;m\in \mathbb{Z}, \end{array}$$

where each *B*_*m*_ is a bounded linear operator on *X* such that *A*_*m*_ + *B*_*m*_ is invertible, *m* ∈ *ℤ*. The corresponding evolution operator of (12) is given by

(13) $$\begin{array}{c} \hat{𝒜}\left(m,n\right)=\{\begin{array}{cc} \left(A_{m-1}+B_{m-1}\right)\cdots \left(A_{n}+B_{n}\right), & m>n,\\ Id, & m=n,\\ {\left(A_{m}+B_{m}\right)}^{-1}\cdots {\left(A_{n-1}+B_{n-1}\right)}^{-1}, & m<n. \end{array} \end{array}$$

**Lemma**

Let *a*, *D*, *δ* > 0. If

(14) $$\begin{array}{c} 2\delta D\frac{1+e^{-a}}{1-e^{-a}}<1, \end{array}$$

then the constants

(15) $$\begin{array}{c} {\tilde{a}}_{1}=-\ln\left(\cosh a-\sqrt{\cosh^{2}a-\left(1+2\delta D\sinh a\right)}\right),\\ {\tilde{a}}_{2}=\ln\left(\cosh a+\sqrt{\cosh^{2}a-\left(1+2\delta D\sinh a\right)}\right) \end{array}$$

are well defined and positive. Moreover, ${\tilde{a}}_{1}\leq {\tilde{a}}_{2}$.

**Proof**

We first prove that

(16) $$\begin{array}{c} \cosh^{2}a-\left(1+2\delta D\sinh a\right)>0. \end{array}$$

Indeed, since

(17) $$\begin{array}{c} \frac{1-e^{-a}}{1+e^{-a}}<\frac{e^{a}-e^{-a}}{2}, \end{array}$$

we have that 2*δD* < sinh⁡*a*, which is equivalent to

(18) $$\begin{array}{c} 2\delta D<\frac{\cosh^{2}a-1}{\sinh a}. \end{array}$$

Hence, cosh⁡^2^ *a* − (1 + 2*δD*sinh⁡*a*) > 0. Now it is easy to see that

(19) $$\begin{array}{c} \cosh a\pm \sqrt{\cosh^{2}a-\left(1+2\delta D\sinh a\right)}>0 \end{array}$$

and thus the constants ${\tilde{a}}_{1}$ and ${\tilde{a}}_{2}$ are well defined. To show that ${\tilde{a}}_{1}>0$, we need to prove that

(20) $$\begin{array}{c} \cosh a-\sqrt{\cosh^{2}a-\left(1+2\delta D\sinh a\right)}<1. \end{array}$$

An easy computation shows that this is equivalent to (*e* ^*a*^ − 1)(1 − *e* ^−2*a*^) > 0, which obviously holds. On the other hand, since 1 − cosh⁡*a* < 0, we obtain that

(21) $$\begin{array}{c} \sqrt{\cosh^{2}a-\left(1+2\delta D\sinh a\right)}>1-\cosh a \end{array}$$

and thus ${\tilde{a}}_{2}>0$. Since

(22) $$\begin{array}{c} {\tilde{a}}_{2}={\tilde{a}}_{1}+\ln\left(1+2\delta D\sinh a\right), \end{array}$$

we have that ${\tilde{a}}_{1}\leq {\tilde{a}}_{2}$. This completes the proof of the lemma.

We set

(23) $$\begin{array}{c} {\tilde{D}}_{1}=\frac{D}{1-\delta De^{-a}/\left(1-e^{-(a+{\tilde{a}}_{1})}\right)},\\ {\tilde{D}}_{2}=\frac{D}{1-\delta De^{a}/\left(e^{a+{\tilde{a}}_{2}}-1\right)}. \end{array}$$

The roughness of nonuniform exponential dichotomy on both *ℤ*_+_ and *ℤ*_−_ was studied in [4]. The authors obtained the following results.

**Theorem 7 (see [4, Theorem 6])**

If (3) has a nonuniform exponential dichotomy on *ℤ*_+_ with *ɛ* < *a* and the sequence (*B*_*m*_)_*m*≥0_ satisfies

(24) $$\begin{array}{c} ||B_{m}||\leq \delta e^{-2ɛ(m+1)},\;m\geq 0, \end{array}$$

for some sufficiently small *δ* > 0, then the perturbed equation (12) has a nonuniform exponential dichotomy with the constants *a*, *D*, and *ɛ* replaced by $\tilde{a}={\tilde{a}}_{1}$, $\tilde{D}=4D\max\left\{{\tilde{D}}_{1},{\tilde{D}}_{2}\right\}$, and $\tilde{ɛ}=2ɛ$.

**Theorem 8 (see [4, Theorem 7])**

If (3) has a nonuniform exponential dichotomy on *ℤ*_−_ with *ɛ* < *a* and the sequence (*B*_*m*_)_*m*≤0_ satisfies

(25) $$\begin{array}{c} ||B_{m}||\leq \delta e^{-2ɛ|m+1|},\;m\leq 0, \end{array}$$

for some sufficiently small *δ* > 0, then the perturbed equation (12) has a nonuniform exponential dichotomy with the constants *a*, *D*, and *ɛ* replaced by $\tilde{a}={\tilde{a}}_{1}$, $\tilde{D}=4D\max\left\{{\tilde{D}}_{1},{\tilde{D}}_{2}\right\}$, and $\tilde{ɛ}=2ɛ$.

We point out that the assumption *ɛ* < *a* in the theorems above is not a restrictive one since the notion of nonuniform exponential dichotomy is apparently motivated by ergodic theory which states that the nonuniform part in the dichotomies of the “most” equations is arbitrarily small (is as small as desired in comparison to the Lyapunov exponents).

In the theorem below, we establish the roughness of (*ℤ*_+_, *ℤ*_−_)-nonuniform exponential dichotomy.

**Theorem 9**

If the difference equation (3) has a (*ℤ*_+_, *ℤ*_−_)-nonuniform exponential dichotomy with *ɛ* < *a* and the sequence (*B*_*m*_)_*m*∈*ℤ*_ satisfies

(26) $$\begin{array}{c} ||B_{m}||\leq \delta e^{-2ɛ|m+1|},\;\forall m\in \mathbb{Z}, \end{array}$$

for some sufficiently small *δ* > 0, then the perturbed equation (12) also has a (*ℤ*_+_, *ℤ*_−_)-nonuniform exponential dichotomy.

**Proof**

We set

(27) $$\begin{array}{c} \Delta_{+}=\left\{\left(m,n\right)\in {\mathbb{Z}}_{+}\times {\mathbb{Z}}_{+}\;|\;m\geq n\right\}. \end{array}$$

Consider the Banach space

(28) $$\begin{array}{c} {𝒞}_{+}=\left\{U:\Delta_{+}⟶ℬ\left(X\right)\;|\;\underset{(m,n)\in \Delta_{+}}{\sup}e^{-ɛn}||U\left(m,n\right)||<\infty \right\} \end{array}$$

with the norm

(29) $$\begin{array}{c} ||U||=\underset{\left(m,n\right)\in \Delta_{+}}{\sup}e^{-ɛn}||U\left(m,n\right)||. \end{array}$$

According to Lemma 1 in [4], the equation

(30) $$\begin{array}{c} Z_{m+1}=\left(A_{m}+B_{m}\right)Z_{m},\;m\geq n\geq 0, \end{array}$$

has a unique solution *U*_+_ ∈ *𝒞*_+_ satisfying

(31) U+(m,n)=𝒜(m,n)Pn++∑k=nm−1𝒜(m,k+1)Pk+1+BkU+(k,n) −∑k=m∞𝒜(m,k+1)Qk+1+BkU+(k,n),

for all (*m*, *n*) ∈ Δ_+_. Moreover, from the proof of Lemma 19 in [4], we can consider the estimate

(32) $$\begin{array}{c} ||U_{+}\left(m,0\right)||\leq {\tilde{D}}_{1}e^{-{\tilde{a}}_{1}m},\;\text{for}\;m\geq 0. \end{array}$$

We set

(33) $$\begin{array}{c} {\hat{P}}_{m}^{+}=\hat{𝒜}\left(m,0\right)U_{+}\left(0,0\right)\hat{𝒜}\left(0,m\right),\;\text{for}\;m\geq 0. \end{array}$$

By Lemma 2 in [4], we have that ${\hat{P}}_{m}^{+}$ is a projection and

(34) $$\begin{array}{c} \hat{𝒜}\left(m,n\right){\hat{P}}_{n}^{+}={\hat{P}}_{m}^{+}\hat{𝒜}\left(m,n\right),\;\text{for}\;m,n\geq 0. \end{array}$$

We denote by ${\hat{Q}}_{m}^{+}=Id-{\hat{P}}_{m}^{+}$ the complementary projection of ${\hat{P}}_{m}^{+}$, *m* ≥ 0. Easy computation shows that

(35) $$\begin{array}{c} P_{0}^{+}{\hat{P}}_{0}^{+}=P_{0}^{+},\;\;Q_{0}^{+}{\hat{Q}}_{0}^{+}={\hat{Q}}_{0}^{+}. \end{array}$$

Indeed, we have

(36) P0+P^0+=P0+U+(0,0)=P0+(P0+−∑k=0∞𝒜(0,k+1)Qk+1+BkU+(k,0))=P0+.

On the other hand, using the same arguments as in Lemma 1 in [4], we prove that

(37) $$\begin{array}{c} {\hat{P}}_{0}^{+}P_{0}^{+}={\hat{P}}_{0}^{+}. \end{array}$$

For this, we consider the Banach space

(38) $$\begin{array}{c} {𝒞}_{+}^{0}=\left\{U:{\mathbb{Z}}_{+}⟶ℬ\left(X\right)\;|\;\underset{m\geq 0}{\sup}||U\left(m\right)||<\infty \right\} \end{array}$$

with the norm

(39) $$\begin{array}{c} ||U||=\underset{m\geq 0}{\sup}||U\left(m\right)||. \end{array}$$

The operator *L*_+_^0^ : *𝒞*_+_^0^ → *𝒞*_+_^0^, defined by

(40) (L+0U)(m)=𝒜(m,0)P0+ +∑k=0m−1𝒜(m,k+1)Pk+1+BkU(k) −∑k=m∞𝒜(m,k+1)Qk+1+BkU(k),

is a contraction on *𝒞*_+_^0^. Thus, *L*_+_^0^ has a unique fixed point *U*_+_^0^ ∈ *𝒞*_+_^0^. We observe that

(41) $$\begin{array}{c} U_{+}^{0}\left(m\right)=U_{+}\left(m,0\right),\;\text{for}\;m\geq 0. \end{array}$$

Also, we obtain that

(42) U+(m,0)P0+=𝒜(m,0)P0+ +∑k=0m−1𝒜(m,k+1)Pk+1+BkU+(k,0)P0+ −∑k=m∞𝒜(m,k+1)Qk+1+BkU+(k,0)P0+,

for *m* ≥ 0. Setting *U*(*m*) = *U*_+_(*m*, 0)*P*_0_^+^, for *m* ≥ 0, we have that *U* ∈ *𝒞*_+_^0^ and *L*_+_^0^ *U* = *U*. Therefore, *U* must coincide with *U*_+_^0^. This is equivalent to

(43) $$\begin{array}{c} U_{+}\left(m,0\right)P_{0}^{+}=U_{+}\left(m,0\right),\;\forall m\geq 0. \end{array}$$

In particular, for *m* = 0, we obtain the desired identity (37), which implies that

(44) $$\begin{array}{c} {\hat{Q}}_{0}^{+}Q_{0}^{+}=Q_{0}^{+}. \end{array}$$

The same arguments hold for *J* = *ℤ*_−_. For a more thorough approach, we include the proofs. We set

(45) $$\begin{array}{c} \Delta_{-}=\left\{\left(m,n\right)\in {\mathbb{Z}}_{-}\times {\mathbb{Z}}_{-}\;|\;m\leq n\right\}. \end{array}$$

Consider the Banach space

(46) $$\begin{array}{c} {𝒞}_{-}=\left\{U:\Delta_{-}⟶ℬ\left(X\right)\;|\;\underset{(m,n)\in \Delta_{-}}{\sup}e^{-ɛ|n|}||U\left(m,n\right)||<\infty \right\} \end{array}$$

with the norm

(47) $$\begin{array}{c} ||U||=\underset{(m,n)\in \Delta_{-}}{\sup}e^{-ɛ|n|}||U\left(m,n\right)||. \end{array}$$

According to Lemma 10 in [4], the equation

(48) $$\begin{array}{c} Z_{m+1}=\left(A_{m}+B_{m}\right)Z_{m},\;\;m\leq n\leq 0, \end{array}$$

has a unique solution *U*_−_ ∈ *𝒞*_−_ satisfying

(49) U−(m,n)=𝒜(m,n)Qn− −∑k=mn−1𝒜(m,k+1)Qk+1−BkU−(k,n) +∑k=−∞m−1𝒜(m,k+1)Pk+1−BkU−(k,n).

Furthermore, from the proof of Lemma 19 in [4], we have that

(50) $$\begin{array}{c} ||U_{-}\left(m,0\right)||\leq {\tilde{D}}_{2}e^{{\tilde{a}}_{2}m},\;\text{for}\;m\leq 0. \end{array}$$

Setting

(51) $$\begin{array}{c} {\hat{Q}}_{m}^{-}=\hat{𝒜}\left(m,0\right)U_{-}\left(0,0\right)\hat{𝒜}\left(0,m\right),\;\text{for}\;m\leq 0, \end{array}$$

by Lemma 11 in [4], we deduce that ${\hat{Q}}_{m}^{-}$ is a projection and

(52) $$\begin{array}{c} \hat{𝒜}\left(m,n\right){\hat{Q}}_{n}^{-}={\hat{Q}}_{m}^{-}\hat{𝒜}\left(m,n\right),\;\text{for}\;m,n\leq 0. \end{array}$$

We denote by ${\hat{P}}_{m}^{-}=Id-{\hat{Q}}_{m}^{-}$ the complementary projection of ${\hat{Q}}_{m}^{-}$, *m* ≤ 0. Since

(53) Q0−Q^0−=Q0−U−(0,0)=Q0−(Q0−+∑k=−∞−1𝒜(0,k+1)Pk+1−BkU−(k,0))=Q0−,

we obtain that

(54) $$\begin{array}{c} Q_{0}^{-}{\hat{Q}}_{0}^{-}=Q_{0}^{-}. \end{array}$$

Moreover, we have that

(55) $$\begin{array}{c} P_{0}^{-}{\hat{P}}_{0}^{-}={\hat{P}}_{0}^{-}. \end{array}$$

Using the same arguments as in Lemma 10 in [4], we prove that

(56) $$\begin{array}{c} {\hat{Q}}_{0}^{-}Q_{0}^{-}={\hat{Q}}_{0}^{-}. \end{array}$$

Indeed, if we consider the Banach space

(57) $$\begin{array}{c} {𝒞}_{-}^{0}=\left\{U:{\mathbb{Z}}_{-}⟶ℬ\left(X\right)\;|\;\underset{m\leq 0}{\sup}||U\left(m\right)||<\infty \right\} \end{array}$$

with the norm

(58) $$\begin{array}{c} ||U||=\underset{m\leq 0}{\sup}||U\left(m\right)||, \end{array}$$

then the operator *L*_−_^0^ : *𝒞*_−_^0^ → *𝒞*_−_^0^, defined by

(59) (L−0U)(m)=𝒜(m,0)Q0− −∑k=m−1𝒜(m,k+1)Qk+1−BkU(k) +∑k=−∞m−1𝒜(m,k+1)Pk+1−BkU(k),

has a unique fixed point *U*_−_^0^ ∈ *𝒞*_−_^0^, given by

(60) $$\begin{array}{c} U_{-}^{0}\left(m\right)=U_{-}\left(m,0\right),\;\text{for}\;m\leq 0. \end{array}$$

On the other hand, we obtain that

(61) U−(m,0)Q0−=𝒜(m,0)Q0− −∑k=m−1𝒜(m,k+1)Qk+1−BkU−(k,0)Q0− +∑k=−∞m−1𝒜(m,k+1)Pk+1−BkU−(k,0)Q0−,

for *m* ≤ 0. Setting *U*(*m*) = *U*_−_(*m*, 0)*Q*_0_^−^, for *m* ≤ 0, we have that *U* ∈ *𝒞*_−_^0^ and *L*_−_^0^ *U* = *U*. Thus,

(62) $$\begin{array}{c} U_{-}\left(m,0\right)Q_{0}^{-}=U_{-}\left(m,0\right),\;\forall m\leq 0. \end{array}$$

In particular, for *m* = 0, we obtain (56). This clearly leads to

(63) $$\begin{array}{c} {\hat{P}}_{0}^{-}P_{0}^{-}=P_{0}^{-}. \end{array}$$

We set

(64) $$\begin{array}{c} S=Id-\left({\hat{P}}_{0}^{-}-P_{0}^{-}\right)+\left({\hat{P}}_{0}^{+}-P_{0}^{+}\right). \end{array}$$

In the following, we will prove that *S* is an invertible operator. Using (32), we obtain

(65) ||P^0+−P0+||≤∑k=0∞||𝒜(0,k+1)Qk+1+||×||Bk||||U+(k,0)||≤δDD~1e−a∑k=0∞e−(a+a~1)k=δDD~1e−a1−e−(a+a~1).

Similarly, by (50), we have

(66) ||P^0−−P0−||=||Q0−−Q^0−||≤∑k=−∞−1||𝒜(0,k+1)Pk+1−||||Bk||||U−(k,0)||≤δDD~2ea∑k=−∞−1e(a+a~2)k=δDD~2eaea+a~2−1.

Combining the inequalities above, we deduce that

(67) ||S−Id||≤δD(D~1e−a1−e−(a+a~1)+D~2eaea+a~2−1)=2δDDsinh⁡a−δD+sinh⁡2a−2δDsinh⁡a.

Hence, provided that *δ* > 0 is sufficiently small, the operator *S* is invertible. Therefore, we can define the projections

(68) $$\begin{array}{c} {\tilde{P}}_{0}^{+}=SP_{0}^{+}S^{-1},\;\;{\tilde{P}}_{0}^{-}=SP_{0}^{-}S^{-1}. \end{array}$$

Using (9), (37), and (56), we obtain

(69) SP0+=(Id−P^0−+P0−+P^0+−P0+)P0+=(Q^0−+P0−+P^0+−P0+)P0+=Q^0−Q0−P0++P0++P^0+−P0+=Q^0−(Id−P0−)P0++P^0+=P^0+.

Similarly, it follows that $SQ_{0}^{-}={\hat{Q}}_{0}^{-}$. Now we are ready to compute

(70) $$\begin{array}{c} {\tilde{P}}_{0}^{+}{\hat{P}}_{0}^{+}=SP_{0}^{+}S^{-1}{\hat{P}}_{0}^{+}={\hat{P}}_{0}^{+}P_{0}^{+}={\hat{P}}_{0}^{+}. \end{array}$$

Setting

(71) $$\begin{array}{c} {\tilde{Q}}_{+}\left(0\right)=Id-{\tilde{P}}_{0}^{+},\\ {\tilde{Q}}_{-}\left(0\right)=Id-{\tilde{P}}_{0}^{-}, \end{array}$$

and using similar arguments, we have

(72) $$\begin{array}{c} {\hat{P}}_{0}^{+}{\tilde{P}}_{0}^{+}={\tilde{P}}_{0}^{+},\\ {\tilde{Q}}_{0}^{-}{\hat{Q}}_{0}^{-}={\hat{Q}}_{0}^{-},\\ {\hat{Q}}_{0}^{-}{\tilde{Q}}_{0}^{-}={\tilde{Q}}_{0}^{-}. \end{array}$$

Repeating the arguments in the proofs of Theorems 7 and 8, the above equalities prove that the perturbed equation has a nonuniform exponential dichotomy on both *ℤ*_+_ and *ℤ*_−_ with projections

(73) $$\begin{array}{c} {\tilde{P}}_{m}^{+}=\hat{𝒜}\left(m,0\right){\tilde{P}}_{0}^{+}\hat{𝒜}\left(0,m\right),\\ {\tilde{P}}_{m}^{-}=\hat{𝒜}\left(m,0\right){\tilde{P}}_{0}^{-}\hat{𝒜}\left(0,m\right), \end{array}$$

*m* ∈ *ℤ*. Furthermore, since

(74) $$\begin{array}{c} {\tilde{P}}_{m}^{+}{\tilde{P}}_{m}^{-}={\tilde{P}}_{m}^{-}{\tilde{P}}_{m}^{+}={\tilde{P}}_{m}^{+}, \end{array}$$

for every *m* ∈ *ℤ*, we obtain the desired statement.

The previous result is a discrete version of Theorem 5 in [11] in the general context of nonuniform exponential dichotomies in infinite-dimensional spaces.
